# Acceptability of Salt Fluoridation in a Rural Latino Community in the United States: An Ethnographic Study

**DOI:** 10.1371/journal.pone.0158540

**Published:** 2016-07-08

**Authors:** Judith C. Barker, Claudia Guerra, M. Judy Gonzalez-Vargas, Kristin S. Hoeft

**Affiliations:** 1 Department of Anthropology, History & Social Medicine, University of California San Francisco, San Francisco, CA, United States of America; 2 Department of Pediatrics and Department of Preventive & Restorative Dental Sciences, University of California San Francisco, San Francisco, CA, United States of America; 3 Helen Diller Family Comprehensive Cancer Center, Community Education & Outreach/Pasick Research Group, University of California San Francisco, San Francisco, CA, United States of America; 4 Institute for Health Policy Studies, University of California San Francisco, San Francisco, CA, United States of America; 5 Center to Address Children’s Oral Health Disparities, University of California San Francisco, San Francisco, CA, United States of America; University of Washington, UNITED STATES

## Abstract

Compared to other population groups in the United States, caries (tooth decay) is a disproportionately prevalent disease among Latino populations, especially among low-income and rural sub-groups and children under five years of age. Fluoride is a primary preventive for caries. While water fluoridation is a major and effective public health means for delivering fluoride on a mass scale, it does not reach many rural areas or population groups such as Latinos who eschew drinking water from municipal sources. This study examines the acceptability to such groups of salt fluoridation, an alternate means of delivering fluoride long used on a global scale. An ethnographic study in California’s rural Central Valley was performed. Thirty individual interviews and 5 focus groups (*N* = 61) were conducted in Spanish to investigate low-income Latino migrant caregivers’ experiences, views and understandings of domestic salt, oral health, caries prevention and fluoride. Audio data were transcribed, translated, coded and thematically analyzed. Table salt was readily available and frequently consumed. Both adult and child daily sodium consumption was high. Despite a general feeling that it was good, and present in dentifrices or dietary supplements, most participants had little knowledge about fluoride. Concerns were raised about cardio-vascular and other possibly deleterious effects if an increase in salt consumption occurred because fluoridated salt was viewed as having ‘extra’ benefits. Once informed about fluoride’s safety and role in caries prevention, most participants expressed willingness to use fluoridated salt, especially if it benefitted children. Reassurance about its safety and benefits, and demonstration of its taste, were important aspects of acceptance. Taste was paramount. Participants would not consume more fluoridated salt than their current salt as that would result in unpleasant changes in food flavor and taste. While salt fluoridation is acceptable, the feasibility of producing and distributing fluoridated salt in the United States is, however, complex and challenging.

## Introduction

Fluoridation of public water supplies is the primary public health approach to caries prevention in the United States (US), reducing dental decay between 20%-40% [1]. Water fluoridation, hailed as one of the 10 great successes in public health in the 20^th^ Century, involves the addition of fluoride to municipal water supplies to reach an optimal concentration of 0.7 ppm F [2]. The amount added is adjusted for each location depending on the natural level of fluoride in the water in that locale. In 2012, more Californians have access to fluoridated water than people in any other state: 24,215,234 or 63.7% of the total population. Yet more Californians also lacked access to fluoridated water than in any other state [3]. Fluoridation of municipal water supplies is sometimes not economically or technically feasible, especially for small rural communities where the cost per person of fluoridating water can be many times that of the cost in more populous urban areas [3–7].

In many places in the United States, both urban and rural, there is mistrust of public water supplies, tap water avoidance and increased use of commercially filtered or bottled water [8–15]. Commercial water services often use reverse osmosis in their filtration process thereby eliminating fluoride from the water. Most bottled water does not contain an optimal level of fluoride for caries prevention [2]. Thus any population that relies heavily on commercial or bottled water for domestic purposes significantly reduces its exposure to a proven caries preventive [5, 8–10].

Several studies have shown that Latino populations strongly prefer to consume commercial filtered or bottled water rather than available municipal supplies [10–15]. Yet exposure to optimal levels of fluoride could benefit these communities. Children in many Latino families in the US, especially those in low-income families or families living in rural areas or engaged in farm work, are at very high risk for developing early childhood caries (ECC), and of suffering immediate as well as life-long consequences, such as infections, malocclusions, speech and communication difficulties, lost days from school, as well as pain [16–26]. Latino adults in these same circumstances can also experience disproportionately high rates of untreated caries and the consequences thereof, such as pain and tooth loss [16, 19, 22, 27–32].

Despite the financial burden that purchasing water places on low-income families [10], over 95% of respondents in one rural California town bought water from commercial sources [13]. Because of undesirable organoleptic qualities—ie, poor taste, cloudy or discolored appearance, and unpleasant smell—the municipal water supply was widely alleged to be ‘unsafe’ to consume despite its compliance with all federal water quality standards [13]. While the basic concept of water fluoridation was acceptable to this population especially if it could be shown to be beneficial to children’s health, it would not be effective if delivered via the mistrusted and underused municipal water supply.

Topical delivery of fluoride occurs via dentifrices and mouth rinses. Prescription of fluoride drops or tablets to children by medical or dental professionals willing to do so, accomplishes systemic and topical delivery of fluoride [17–18]. Prescription fluoride supplements are designed to be swallowed, sucked or chewed before ingestion, or swished around the mouth for topical effect. Topical delivery of fluoride is widely accepted by low-income, rural and migrant Latino populations [33] but all these methods rely on parents deliberately choosing to use these products for their children. In contrast, public health endeavors that do not rely on the purposeful actions or compliance of individuals have a greater potential for consistency of use and therefore of benefit. The most successful public health interventions, such as water fluoridation [1], are readily available on a mass scale, low cost to consumers and easy to access and use. Rejection of municipal water supplies by Latino populations, suggests that an alternate public health means of fluoridation besides the water supply is warranted.

In the last 100 years, fortification of food through addition of specific micronutrients (e.g., iodide, iron, folic acid, Vitamin A) to common dietary staples, such as salt, cereals, bread, flour, or milk, has become a standard practice worldwide [34]. Food fortification represents an inexpensive to produce, stable, low cost to consumer, and effective means of delivery on a mass scale of substances aimed at controlling specific diseases or conditions (e.g., goitre, anemia, neural tube defects, xerophthalmia, scurvy) [34–38]. In some countries, milk and salt have been investigated and employed to deliver fluoride as a micronutrient aimed at reducing dental caries [39–44].

Salt fluoridation is a well-established public health means for delivery of fluoride. It has been used in some European countries for decades (e.g., in Switzerland since 1955), and increasingly since the early 1990’s in Central and South American countries including Mexico, the major source of Latino migrants into the United States [5, 39–44]. Presently, the number of people worldwide using fluoridated salt has reached approximately 300 million, including 200 million in Latin America and 70–80 million in Europe. Salt fluoridation comprises the addition of a mixture of potassium fluoride (KF) and sodium fluoride (NaF) to domestic or table salt to reach a concentration of 250–300 mg of F/kg salt (i.e, 250–300ppm) [39]. A recent review showed that in some circumstances salt fluoridation was a more cost effective caries preventive for children than either water or milk fluoridation or use of fluoridated mouth rinses [45]. Despite its long use clinically, there is a relatively scant literature on the effectiveness of salt fluoridation in reducing caries incidence or increment. Several meta-analyses of various databases point to few if any RCT investigations or high quality research protocols being used to document well defined clinical outcomes. Nevertheless, a consensus exists that exposure to salt fluoridation prevents more cavities than no such exposure at all [46–51].

Since low-income Latinos in the US especially farmworkers and their families who experience very poor oral health status including disproportionately high rates of early childhood caries (ECC), live in places where fluoridation of water is often not feasible, some other means of fluoridation would be desirable. Salt fluoridation is one possible alternative. Before embarking on any large-scale salt fluoridation project in the US, however, it is first necessary to know if such a public health practice would be acceptable to these communities and under what circumstances. Domestic salt consumption habits, beliefs and preferences of rural Latinos have substantial implications for the success of any possible salt fluoridation project. But these are poorly investigated topics. Much more needs to be known about the multiple cultural, social or structural factors that could reduce or prevent fluoride exposure through salt. Reported here, then, is a case study addressing these social and behavioral issues.

### Overall Goal

The overall goals of this study were to explore and evaluate: (1) whether fluoridated salt would be acceptable to a low-income rural migrant Latino community in the US, and (2) how domestic salt use, cultural beliefs and experiences would help shape and influence acceptability.

Let us be clear. We did not set out to nor did we undertake a dietary survey to determine if current nutrient intake, water consumption or oral health habits resulted in adequate exposure to fluoride. Our concern was solely to identify and understand social and behavioral facilitators and barriers to acceptance of any potential salt fluoridation program. Before any such future program could be implemented, considerable nutritional research would first be necessary to establish current fluoride intake levels for people of all ages in this and similar communities to ensure that over-exposure to fluoride and its consequences would not occur. That is a very different study to this one, far beyond the scope of work reported here.

To address the social and behavioral questions surrounding acceptability of salt fluoridation, we undertook formative qualitative (ethnographic) research of Latino parents with children under age 10 in the same rural community in California's Central Valley where we had previously worked [13, 26, 31–32, 52–55]. Results from our previous work are consistent with those from other studies in general and on oral health, conducted with similar populations in other parts of the United States [56–59].

California is a major agricultural state with high proportions of Latino migrant and non-migrant farmworkers in the population, and so it served as a good place in which to situate the study. Like migrant farmworkers generally, in addition to oral health problems, people in this community experience a range of acute and chronic illnesses, some of which are directly associated with agricultural occupations, some of which may be exacerbated by (excess) dietary consumption of salt [60–65]. Besides oral health problems, other prevalent health conditions include musculoskeletal problems, diabetes, cardio-vascular disorders, skin diseases, depression and other mental health conditions, [60–65].

## Methods

### Study Site

The community’s permanent resident population of around 11,148 is 97 percent Latino-origin, comprising mainly recent and a few second-generation immigrants, mostly from Mexico and other Latin American countries [64]. Non-livestock agriculture is the main economic enterprise, especially the growing of cantaloupes, tomatoes, asparagus, and other vegetables. For the period 2010–2013, which encompasses data collection for this study, approximately half (46%) of the 2, 884 households were at or below the US 2010 federal poverty level [66], partly due to high seasonal unemployment as well as the current drought.

This small city hosts a Federally Qualified Health Center providing primary care and basic medical emergency services, and an attached dental clinic. There are two other private general dentists in this city, and more than a dozen dentists in neighboring communities within a 30-mile radius [52]. The community does not add fluoride to its water supply. In 2006, the natural fluoride level of the water was 0.6ppm on average [13], slightly below the optimal level (0.7ppm). The vast majority of the population (95%), however, reported purchasing filtered or bottled water at $1.00/gallon and not consuming the available tap water for drinking; about half noted they use municipal water when cooking [13].

### Procedures

Primary research questions governing the data gathering activities in this study were:

1. What is the range of beliefs regarding fluoride and salt, and the range of behaviors regarding salt consumption and exposure to fluoride among immigrant and US-born Latino residents, especially but not exclusively those with children aged under ten?
2. Among this population, what knowledge and beliefs exist regarding fluoride’s safety, effectiveness and importance in improving oral health? What is this population’s knowledge of and experience with dental fluorosis or with salt fluoridation elsewhere, such as in Mexico?
3. What is the acceptability to this population of potential fluoride exposure through salt, especially with respect to children? In light of recent public health campaigns about (high) salt intake and cardio-vascular health, what kind of concerns or issues do people in this community have about salt consumption, whether fluoridated and not?

**Fig 1:**
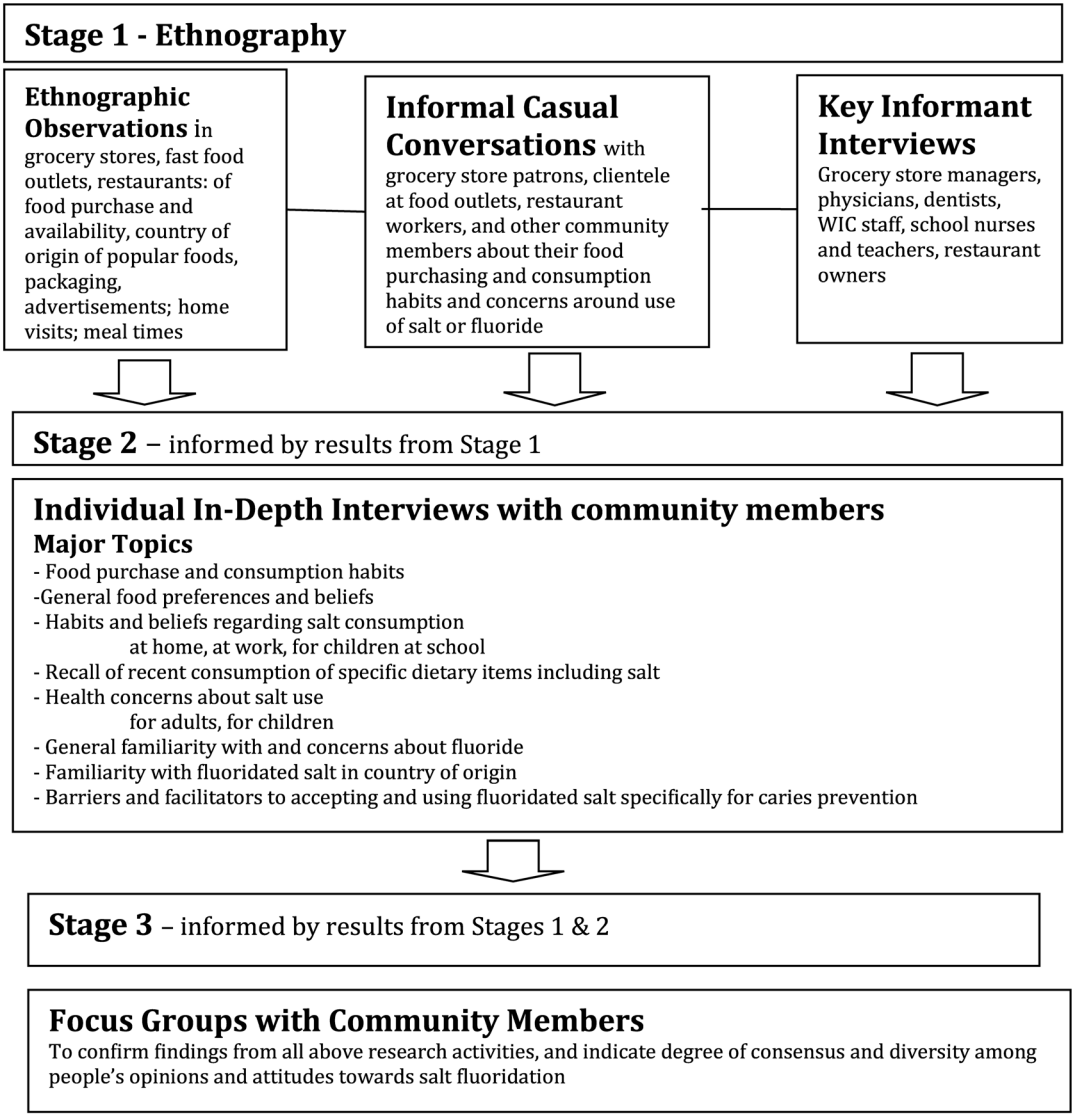
Flow chart showing connections between ethnographic stages.

Given these research questions and overall goals, an ethnographic approach was used to explore this under-studied topic, through use of qualitative interviews, focus groups and observations. Fig 1 (Fig 1) depicts the overall research process through three distinct but connected ethnographic stages.

#### Stage 1

This initial stage consisted of various informal “scene-setting” contextual observations and casual conversations with store patrons, parents with children in playgrounds or at community events, restaurant workers and their clientele. Very broad-ranging but targeted conversations were held with key informants (such as grocery store managers, local dentists, physicians, federal nutrition program [WIC] officials, preschool teachers and so forth). Extensive written field notes kept about all encounters provided valuable additional information on the setting and context as well as non-verbal cues about salt habits (e.g., visible presence of salt shakers on tables). Photographs of store shelves recorded salt marketing displays and availability of salt types. A citywide map was made of the location, size and nature of stores selling general groceries, packaged or processed foods, take-out foods or restaurants serving cooked food on the premises.

#### Stage 2

The semi-structured individual interview guide used in Stage 2 incorporated findings from Stage 1 as well as ideas derived from literature reviews of pertinent topics. Brief focused ethnographic observations were also made in people’s homes during formal interviews. Major questions focused on beliefs about salt, its role in household cooking activities, salt use habits and practices, daily diet, and health of both children and adults. Knowledge, concerns and beliefs regarding fluoride and its relationship to oral and systemic health were also examined along with the acceptability of a salt-based fluoride delivery mechanism. Participants completed a brief questionnaire about their socio-demographic characteristics and self-rated health. Three structured questions on regular salt consumption habits were adapted from the ASA24^™^ dietary recall questionnaire [67]. This instrument is well suited for measuring current dietary intake for specific population groups rather than for individuals, corresponds well to observations of food intake, and has been successfully used previously with Hispanic populations [67].

#### Stage 3

In Stage 3, new participants engaged in focus groups to: (a) confirm findings from the analysis of Stage 2 data, (b) explore in more depth central topics and (c) discuss new topics or questions that emerged from the previous stages. Focus groups are a particularly effective method for developing a deeper understanding of the reasons behind poorly-investigated or understood beliefs or behaviors [68–69], such as salt consumption habits and beliefs. Moreover, their interactive group format encourages a relaxed discussion among marginalized populations such as farmworkers or recent immigrants who may otherwise feel less comfortable expressing their views to outside researchers. Participants completed the same socio-demographic questionnaire as in Stage 2. To better understand what people meant by “usual amount” when discussing their domestic salt use, each individual in the focus groups was asked to place into a 3 liter (12 cup) crock pot the amount of salt they would typically use when cooking 0.5kg (1lb or 2 cups) of (dry) pinto beans. This would produce 5 cups or approximately 10 servings of cooked beans [70]. The quantity of salt each individual placed in the bean pot was then individually collected and weighed.

Study procedures were approved by the University of California San Francisco’s Institutional Review Board (#10–04246), and conformed to the funder’s approved protocol and Clinical Terms of Award. Further, all study procedures were informed by and approved by local community advisory boards. One board comprised a physician’s assistant, dentist, and school nurse, all of whom actively worked in the city and had specific views and concerns about the population’s current salt habits and any potential changes in these. In addition, we met with and received approval for the project from a community advisory board established by our informal research partner, MICASA, a project focused on farmworker environmental/ occupational health conducted through the University of California Davis [64]. This larger group of advisors comprised elected city officials, farm workers, union representatives, agricultural employers, business leaders, a physician, and church and community leaders as well as local citizens. Study findings were made available to these community advisory groups.

All formal study participants (key informants, caregiver interviewees and focus group members) were adults aged 18+ years; each provided written informed consent. Interviews consisted of guided conversations using a semi-structured format with open-ended questions and extensive use of probes. Individual interviews took place in a private location of the interviewee’s choice, usually their home. Each focus group was held in a private meeting room at the study’s field office. Each formal interview or focus group session lasted between 1½ and 2 hours. Key informants were very modestly compensated for their time ($50 donation in their name to a charity of their choice), while interview and focus group participants each received a $30 gift certificate to a local grocery store.

Interviewers were trained, highly experienced bilingual researchers. Apart from a few key informant interviews, each interview or focus group session was conducted in Spanish, digitally audio-taped, and professionally translated and transcribed by a native Spanish speaker. Occasionally participants switched from Spanish to English or vice versa during interviews or group conversations but the bulk of the data was gathered in Spanish. Transcription was checked for completion and accuracy by study staff who back-translated as necessary, and compared transcribed text to the audiotape, noting any difficult to translate segments [71].

Eligible participants in Stage 2 and 3 were adults who self-identified as Latino and as a parent or primary caregiver to a child aged 1–10 years. Participants were recruited in various ways: through personal contact by study staff who visited child-centered sources, such as WIC, preschools and Head Start; renewed contact with respondents in prior studies; Zumba and other exercise classes; or referrals from other participants. Flyers were posted in public sites but did not generate participants. Farmworkers, especially those who were relatively recent arrivals from Mexico or elsewhere in Central America, comprise a hard-to-reach population among whom sampling can be challenging [64]. Four of the five focus groups comprised people solicited through our informal partner, the MICASA research project [64]. Recruiting through this population-based farmworker environmental/ occupational health project that had a carefully enumerated and selected longitudinal cohort of 467 households in the town, enabled us to considerably reduce biases inherent in worksite, school or clinic sampling.

Analysis of how participants gave meaning to their experiences, and how cultural context and local environment shaped salt-related beliefs and behaviors was guided by the broadly social constructivist theoretical approach underpinning this study. This approach relies on data collection and constant comparison of themes presented by participants to develop a conceptual model of participants’ ideas as these emerge directly from the observational and text data [69, 72–74]. This qualitative approach has been successfully used to explain other health-related phenomena that are otherwise poorly-understood. It yields a rich understanding of salt use and acceptability of salt fluoridation. Researchers independently applied two types of codes: a set of *a priori* codes for ideas developed from the existing literature, and a set of codes that encompassed themes emerging from the transcripts. Codes were developed, refined and iteratively applied to the text using QSR International’s qualitative software program NVivo^®^ 10.0. [NVivo qualitative data analysis software; QSR International Pty Ltd. Version 10.0, 2012]. Where appropriate, descriptive statistics were generated using Stata^®^ [Stata Statistical Software: Release 13. College Station, TX: StataCorp LP.; 2013].

Illustrative quotations, typical comments made by respondents, are included in the Results section. This paper reports only on the acceptability of fluoridated salt in this community. Participants self-reported salt use habits are described in more detail elsewhere.

## Results

In this community, salt was a powerful and central component of daily life in both family and work contexts. Salt for domestic use was readily available and widely used. This study specifically excluded consideration of salt used for other purposes, such as water softening. For the purposes of this report also, we use the term ‘sodium’ to mean salt, specifically table salt. While participants generally identified salt as healthy and necessary both to bring out the flavor in food and as a means to manage health when engaged in agricultural fieldwork, they also expressed concerns, particularly about salt’s role in cardiovascular disease. These factors all influenced the potential acceptability of any fluoridated salt product.

### Participant Characteristics

Responses to the questionnaire provided basic insights into interview and focus group participants’ socio- demographics, self-reported health status, and salt habits. The total of 61 participants were predominantly low-income Spanish-speaking residents in the study site, most of whom were engaged in farm work or its associated industries, such as in canning or packing factories or in produce distribution such as in truck driving (details in Table 1).

**Table 1:** Participant Characteristics (N = 61).

	n, % or Mean (±SD)
Caregivers/Individuals Interviewed	n = 30
Focus Group Participants*	n = 31
Gender (female)	87%
Mean Age (years)	41 (±12)
Education (average grade/years completed)	6 ± 4
Nativity	
Mexico	*n* = 35 (57%)
El Salvador	*n* = 19 (31%)
US	*n* = 5 (8%)
Honduras	*n* = 2 (3%)
Years living in U.S. **	17 (± 9)
Predominantly Spanish-speaking at home ***	93%
Speaks English “not well at all” ***	83%
Mean number of children in home	2 (±1.3)
Self or partner involved in farm work	84%
Has health insurance—self only ***	44%
Last medical visit 2+ years ago	10%
Has dental insurance—self only***	18%
Last dental visit 2+ years ago	27%

* One focus group comprised men only (*n* = 5)

** If foreign born

*** Asked of individual interviewees only (n = 27)

### Salt Availability and Use

It was easy to locate and purchase salt in this town that hosted four small- to medium-size full-service grocery stores, none of which was associated with a regional or national chain. In addition, there were 14 mini-mart or small convenience stores (*bodega*s), most offering a limited selection of packaged and fresh food along with household products or hardware items. An inventory was made (name, location, main menu items) of all 19 fast-food outlets, taco trucks, *pupuserias*, and quick service sit-down coffee shops/restaurants that sold prepared food or meals. Conversations were held with store and restaurant managers about their salt purchasing habits and their use of salt in locally made foods.

A few small stores did not sell salt at all, providing only items on the WIC approved food list. Every other retail establishment sold several brands of iodized salt along with smaller quantities of non-iodized salt. Table salt was sold mainly as a fine-grained pouring salt but larger granule sea salt or kosher salt was also available in the larger stores and used in cooking. Most popular with customers was a major US national brand with an easily recognized label, sold in 735 gram (26oz) canisters. Six other brands were available (two of which were imported from Mexico) especially in the small mini-marts or *bodega*s. Both iodized and non-iodized forms of salt were routinely available; it was possible on a few occasions but not routinely to purchase Mexican-origin salt that was both iodized and fluoridated. These packages were labeled in Spanish as containing both iodine and fluoride. Brands and types of packaged salt available in the larger stores were determined by the wholesale distributors rather than by specific request from the store manager. Also available, especially in convenience stores, were small salt-and-pepper shaker combination packs, a popular item in the lunch boxes of fieldworkers.

The volume of salt sales was extremely variable. Larger stores reported generally selling between 10 and 24 canisters per week whereas in the mini-marts and convenience stores, salt sales ranged widely, from one canister per month to two per week. Container labels clearly noted whether or not the salt was iodized in writing (usually in English, which many participants could not read) and by label color and specific visual emblems. Only three of the 28 individuals interviewed about this topic were aware that salt comes in both iodized and non-iodized forms. Two people said they had heard of fluoridated salt. Most did not know why salt contained iodide or fluoride additives. Salt purchases were made mainly by brand loyalty or package recognition or, to a lesser extent, by price. Even though salt was relatively inexpensive, ranging between a low of 59¢ per canister (or when on sale, two cans for $1.00) to a high of $1.49 per kilogram bag, a few participants reported always seeking the cheapest available and stocking up when salt was on sale.

Participants varied greatly in their salt buying habits. The majority (18 of the 30 Stage 2 interviewees, 60%) reported frequently purchasing salt, generally buying a canister 1 to 2 times per month; 4 of these 18 people bought a canister of salt each week. While two people (2 of 30, 7%) reported buying salt as infrequently as once or twice year, the rest (8 of 30) bought 3 to 6 canisters per year. Some of this variability comes from different household sizes, and some may result from people using table salt for purposes other than cooking. For example, field hands reported soaking their feet in warm salted water to soothe tired or painful muscles. We did not ask participants to estimate the proportion of salt that went for food preparation or consumption versus other uses.

The amount of salt purchased depended not just on household size but also on the number of home cooked meals being prepared, the number of people being fed at each meal, and the desired /acceptable taste of the food. Most participants noted that they generally ate at home and helped prepare or cook all (three) daily meals. The vast majority of participants (91%) reported using ordinary table salt rather than any kind of salt substitute. While two-thirds of the sample said they used salt during cooking only, about a half of the participants acknowledged that at least occasionally they also added salt at the table while eating. The mean weight of salt added to bean pot during that exercise was 19.2 grams (± 10.6grams; range = 2.4–60.3 grams; median = 17.4 grams), equivalent to 3.4 teaspoons. This amounts to approximately 2 grams per serving of cooked beans—a very significant proportion of the recommended daily intake of 2,300 milligrams of sodium (about a teaspoon of table salt per day) [75–76]. Men more than women tended to purchase and consume food outside the home. Beans, rice, chicken, soups, bread, pizza, prepared meats (such as hot dogs or chorizo sausage), sodas and sports drinks, were commonly consumed foods. These and other frequently used commercially produced, processed foods, (eg, bouillon cubes, ramen noodles) contain high levels of salt.

#### Sodium and salt

All participants had heard of or read about the term “sodium”, the term generally used in the biomedical literature, on food labels and by health care practitioners. But only some participants were clear on what sodium is, and how it relates to table salt.

***<Interviewer 1>*** *And what do you think sodium is?*

**<Respondent 1>** I don’t know.

***<Interviewer 1>*** *Have you heard that word?*

**<Respondent 4>** Yes.

**<Respondent 7>** Yes.

**<Respondent 6>** Yes, you hear it a lot, but I don’t know what it is.

Participants who knew about sodium gathered their knowledge mainly from reading the information on nutrition labels on packaged foods, taking WIC classes or getting counseling from physicians, nurses or nutritionists, or hearing about it from friends and family members (who, in turn, often learned this from physicians or nutritionists). There was general consensus among participants that sodium and salt are highly related, as explained by this participant: “*they [sodium and salt] are almost like brother*, *I think they come together*.”

Some participants were leery of products that contained ingredients in addition to salt, especially ingredients which were not familiar, as this focus group discussion suggests:

**<Respondent 2>** It [brand of reduced salt or diet salt product] has twenty five percent less salt. But we read it, the whole label, my wife or I read it, but it has more harmful things… the one that has less salt, because it has other amounts of…

***<Interviewer1>*** *So, it has less salt, but it has more of other things?*

**<Respondent2>** Other things.

***<Interviewer1>*** *Okay*.

**<Respondent2>** So it turns out better to use the regular one [salt] than the one that has twenty five percent less salt, because it has… I’m not sure what to call it… additives or I don’t know what they are called, to give more flavor to things

### Fluoride Knowledge and Beliefs

These comments and suspicions about the safety and general merits of substances added to some types of salt, led to discussion about adding fluoride to table salt. Similar to previous reports for this town [13], participants were generally not familiar with fluoride. For those who had heard of fluoride, most got their information from taking their children to the dentist; a few had noticed fluoride listed as an ingredient on toothpaste labels. Most were unaware, however, of its value in dental caries prevention. The few who were familiar with the term claimed it was for cleaning, whitening or protecting the teeth, or for protecting bones. A small number of participants recognized that their children had been prescribed fluoride drops or tablets by dentists or physicians and felt that fluoridated salt would be an easier, cheaper and safer way to provide a caries preventive to their children. This focus group discussion illustrates these points.

***<Interviewer 1>*** *And so now we are going to talk about the salt with fluoride*. *Have you ever heard about salt with fluoride?*

**<Respondent 8>** Salt with fluoride?

***<Interviewer 1>*** *Um-hum*.

**<Respondent 8>** I’ve heard about fluoride, that tooth paste have them right?

***<Interviewer 1>*** *Tooth paste, yes*.

**<Respondent 8>** But not that it’s mixed with salt.

**<Respondent 9>** Not me neither, with water yes, but with salt.

***<Interviewer 1>*** *Or do you know what fluoride is for? Like [G] said it’s what they add to the tooth paste, so for what do you think…?*

**<Respondent 9>** Isn’t it to whiten?

**<Respondent 8>** To whiten your teeth, no?

**<Respondent 2>** To build resistance and to whiten your teeth according to what they say, to have them [teeth] healthier.

***<Interviewer 1>*** *And where did you hear that?*

**<Respondent 2>** At the same clinic here.

**<Respondent 4>** At the dentists.

**<Respondent 2>** At the dentist with my son, because I have a son who has cavities and they are giving him pills, but they are fluoride pills and they told me it was for him to chew them so that the gums could absorb it.

***<Interviewer 1>*** *Oh*.

**<Respondent 2>** If the gums don’t absorb it when he [child] chews, they [pills] are fluoride he [dentist] said, if they [gums] don’t absorb it, and he [child] swallows it, the body will absorb it anyways, like a vitamin, but they are special for his teeth. The fluoride is stronger.

***<Interviewer 1>*** *Oh. Have you heard anything else?*

**<Respondent 2>** There at the dentist is where I’ve heard the rest.

**<Respondent 6>** Yes, me too.

***<Interviewer 1>*** *Yes? Also about the pills? Or about something else?*

**<Respondent 4>** No, just about the tooth paste and all of that and for the cavities, the fluoride.

***<Interviewer 1>*** *For cavities?*

**<Respondent 4>** To clean them, like when the kids have cavities, tartar, all of that cleans it.

**<Respondent 2>** Yes.

Only one individual, a recent migrant from Mexico, recognized or mentioned knowing about fluoridated salt even though it is readily available and widely used there. This participant had been a cheese maker and knew how iodized and fluoridated salt affected the preparation and appearance of prepared foods. Another woman, Respondent 2 in the focus group excerpt below, might have been referring to fluoridated salt when she explained that children needed salt. She comes from Michoacan, a Mexican state where salt fluoridation is commonly used as a caries preventive [77]. What this conversation mainly reveals, however, is that while she remembers being taught that salt was good for her children, she does not remember the exact reasons why.

**<Respondent 4>** Salt is salt, and it affects you all the time.

***<Interviewer 1>*** *Or if you start to eat too much salt when you are an adult?*

**<Respondent 4>** I feel that the salt is bad.

**<Respondent 2>** When the kids are small, like I told you, they need it, if they eat it in excess since they’re small, well it’s going to affect them, but if they eat the salt with moderation because they need it, then I don’t think is harmful. Until a certain age when they begin to grow if they continue to eat too much salt, then it starts to affect them, but kids a little bit of salt that they eat, that’s good for them. Because there in Mexico they tell us **‘**Kids need to eat salt in their things because it’s good for them, they need it.’ I don’t remember what the salt had which is good for them. And that’s why I say that as long as the kids are fine, are developing…

***<Interviewer 1>*** *Which part of Mexico, did you say?*

**<Respondent 2>** Michoacan, there, I’m from the central Michoacan. In the health center that’s where they told us. They told us that the kids needed to, how do I tell you? have to eat salt. They gave us talks. They have to eat salt because they need it, it is good for them for their gums, their teeth, all of that. That’s why I don’t think, unless here they say no, but they said that over there.

### Acceptability of Fluoridated Salt

Most people initially said they would not use fluoridated salt until they better understood what fluoride did and why it was important. They were adamant about their need to be clearly and completely informed and assured first of its taste and safety, especially with respect to their children’s health. They also pointed to physicians, dentists or nurses as trusted sources of information who would provide them with reliable, authoritative information about fluoride.

One study participant noted:

***<Interviewer>*** *Have you ever seen one of these containers that says salt with fluoride?*

**<Respondent>** No, or I haven’t paid attention.

***<Interviewer>*** *Would you grab one of these containers?*

**<Respondent>** Not until I was sure what benefits it has.

while another participant commented:

***<Interviewer>*** *Or what type of information would you need in order to be interested in grabbing a container that said salt with fluoride?*

**<Respondent>** Well, if I was better informed I would take it, that’s why I’m used to taking what I already know. That’s why I say I never change seasonings; look here it has one, because I already know these ones.

Yet another stated clearly:

**<Respondent>** Well, depends on what they say, that that salt is good for your body that it doesn’t contain, well that it doesn’t harm the bones, things like that, well people might get excited to buy it.

A fourth interviewee lays out her safety concerns in even more detail:

***<Interviewer>*** *So what would convince you to buy it*? *What would the tin have to have or…*

**<Respondent>** To know what it contains, to know what the salt would contain, if it doesn’t affect the bones, or teeth or the ears, the hair, have you seen how many lose their hair? So that is why I tell you, you heard something mentioned, but if you don’t know what it contains then why would you buy it?

After the interviewers provided information and answered their questions about fluoride’s purpose and safety, most respondents reported a strong inclination to accept fluoridated salt if it were available. For participants with young children, the major incentive for acceptance was the benefit fluoride would confer on their children’s health, specifically their oral health. In answer to the question “*Who would benefit the most by having access to fluoridated salt*?” more often than not the first answer was ‘the children.’

***<interviewer>*** *And would you use this salt with fluoride here with your family?*

**<respondent>** Yes.

***<interviewer>*** *Why?*

**<respondent>** Well, because of the fluoride, too. So that it’d help to strengthen my children’s teeth.

But when probed further, the majority said that everyone would benefit from fluoridated salt, children, adults and seniors. One interviewee said: “*Whoever has teeth*, *evenly*, *because that doesn’t help those of us who don’t have teeth*. *…”* Another participant expanded on this theme *“Because old people*, *children*, *adults… we all have teeth*. *We all have caries and we all lose everything*, *our teeth*. *Well*, *yes*. *But for children*, *to protect them*, *to start protecting them*, *right*?*”*

#### Concerns about adult health and fluoridated salt use

Participants, however, expressed concerns over the possible impact of fluoridated salt on adult health and well-being. High on the list of serious adult health problems prevalent in the community and recognized by participants were diabetes and high blood pressure. Excessive salt intake was clearly identified as a contributing factor, and was linked with eating “junk food”, processed foods, insufficient exercise, difficult working conditions, and general stress especially around financial instability.

***<Interviewer>****“ what if you use too much salt?”*

**<Respondent 2>** “Like the lady was saying—heart attacks, high blood pressure, blood problems, as they are called. There would be a lot of problems. If you use the regular amount or less [of salt], then there would be less risks.”

Local health care providers also expressed concern about possible over-consumption of fluoridated salt. They feared people might be tempted to use more fluoridated salt than non-fluoridated salt because of the perceived health benefits of fluoridated salt. When explicitly asked, all respondents rejected the idea that they would consume more fluoridated salt. Participants explained that using a greater quantity of salt than at present would make food taste unpleasant, be too salty. In essence, then, salt was viewed as a necessary substance, capable of enhancing flavor and maintaining health but only so long as it was not consumed in excess (according to their definitions). Two women interviewees explain. The first says:

***<Interviewer>*** *And do you think you would use it the same way as you use the other salt normally or would you use more, or less?*

**<Respondent>** No, the same. Like they say, a little of the good thing.

***<Interviewer>*** *How do you think you would use that [fluoridated] salt? The same as this on [points to current national brand canister of table salt], more than this one, less than this one?*

**<Respondent>** Well, I think the same

The second respondent endorses these sentiments:

***<Interviewer>*** *Okay. And how do you think you would use it? The same like the normal salt you already use…?*

**<Respondent>** Normally, um-hum.

***<Interviewer>*** *Or would you use more, would you use less?*

**<Respondent>** No, the same.

### Acceptability to Families

Besides the benefit conferred, other factors played key roles in acceptability. In relation to currently available table salt, fluoridated salt also had to look and taste the same, perform in the same fashion during cooking, be as easily obtained in the same grocery stores, and have at best a very modest cost increase Most participants reported that they would seriously consider paying a little more for fluoridated salt if it were safe, beneficial to their children’s or grandchildren’s teeth and had the same taste qualities as currently available table salt. Taste was the paramount consideration.

When asked to predict its acceptability to their families, it became very clear that women who cook are the primary decision-makers, controllers and overseers of family diet and health. For the most part, women in the study said that if fluoridated salt tasted good, they would accept fluoridated salt, as the following conversations reveal.

**<Respondent 6>** Oh, okay, well then I would try it to see how it went with the ‘lawyers’ I have.

***<Interviewer 1>*** *If they like it or not?*

**<Respondent 6>** I don’t have lawyers but it’s as if I had them.

***<Interviewer 1>*** *Oh*.

**<Respondent 5>** I think…

**<Respondent 6>** ‘It has too much salt, it has too much fat, it has too much this.’ And I tell them ‘And it also has too much “*madre*” as well. If you want to eat it, I’m sure you’ll eat it’

***<Interviewer 1>*** *So it depends a lot on the taste?*

**<Respondent 6>** Yes, for me.

***<Interviewer 1>*** *The taste? Okay. And what do you think?*

**<Respondent 5>** I would also use it but I would make a meal, if it was in the morning.

***<Interviewer 1>*** *Um-hum*.

**<Respondent 4>** I’m going to use it when I make my breakfast, lunch and I’m not going to tell them anything. I’m the one who is going to know if I’m going to add it, so I’m going to see if anyone says…

***<Interviewer 1>*** *So see if there is a complaint*.

**<Respondent 4>** To see if there is one person who notices the difference right? ‘What did you add today to the breakfast that it’s more delicious?’ Or something. If it’s just like ‘Oh my stomach hurts!’ Or I don’t know something like that that they mention then perhaps it’s because of that. But I’m the one who is going to think and then I’m going to explain to them what it was, what it is.

Women in another focus group had a similar discussion:

***<Interviewer 1>*** *…. So first you would try it?*

**<Respondent 4>** First I would try it one day and then…

**<Respondent 1>** I think I would do the same.

***<Interviewer 1>*** *Really?*

**<Respondent 1>** Because I don’t know, I’ve never tried that brand or anything but if it’s for my health and it is good for my family, I would use it.

***<Interviewer 1>*** *Really? And it depends on the taste?*

**<Respondent 1>** Yes, aha.

***<Interviewer 1>*** *If the flavor changes, then no?*

**<Respondent 1>** No, if I see that’s good for my family I would use it.

***<Interviewer 1>*** *Oh*.

**<Respondent 5>** If it’s good for my health, I would use it.

**<Respondent 4>** They [health experts] should make it known first ‘Look there is this, it has this, it has this.’ And well if they say “it’s good for your health” then you have to take it.

**<Respondent 3>** I would use it the same way as the normal one because my family doesn’t even realize what brands I use of anything.

**<Respondent 2>** It’s the same in my case.

***<Interviewer 1>*** *It’s the same thing, no?*

**<Respondent 3>** Yes.

**<Respondent 2>** I’m the one who cooks and that’s it. I would add it and I wouldn’t tell them.

**<Respondent 5>** Yes, but they realize what we use. Even though they don’t cook, but they see…

***<Interviewer 1>*** *The tin*.

**<Respondent 5>** What we take. Because they are grownups how are they going to say ‘no’, or ‘use another one’. Because even though they don’t cook, they say ‘Oh my mom uses this one.’ Or ‘She always takes this one.’ Like that, so that right?

## Discussion

In this study, salt was seen as a vital component of food and nutrition, an essential ingredient in the cooking, stabilizing and preserving of foods as well as for bringing out flavor [75]. Salt was viewed as an essential staple necessary to sustain life. Domestic salt, both with and without iodide added, is readily available in grocery stores and is a common household item purchased and used regularly. The amount of salt purchased annually varies widely, depending on family/household size, number of meals eaten at home each day, and cost. Neither salt used in home cooking nor added at table was thought notable or even problematic if used in moderation. It was claimed that fluoridated salt would not be consumed in greater quantities even though it may be viewed as better for one’s health than current table salt because participants felt strongly that they would be guided by taste.

In this convenience sample of predominantly migrant, low-income Hispanic parents or primary caregivers of young children in a rural setting, fluoridated salt would be an acceptable means of delivering a proven preventive for caries—but only under certain circumstances. Fluoridated salt would be acceptable provided that community members had first received information from trusted, authoritative sources about the safety and benefits of fluoride, that table salt containing sodium fluoride as an additive was conveniently available at approximately the same cost, and that it tasted the same as non-fluoridated salt. This finding is consistent with other reports in the literature which show that taste is paramount: food additives will not be accepted if they are perceived as changing the flavor, the taste of the food [38, 75–76].

Probable prior exposure of these respondents to fluoridated salt in their home countries was not broadly recognized. On average, however, Hispanic migrants in this study, who comprised the majority of the sample, were 41 years of age and had lived in the US for over 15 years. Therefore they were likely not adults when fluoridated salt was being introduced in the 1980’s and 1990’s to Mexico and other Central American countries [39, 41–44, 77]. As children or young adults, these study participants were likely unaware of the exact nature of the salt consumed in their households.

Women—the family cooks—were revealed to be key gate-keepers, not just for overall family health, especially children’s health, but also for the purchase and consumption of food, and the introduction of new dietary items. The content of the daily diet and consumption habits are largely under the control of the adult women in the household. They were the initial arbiters for acceptance. Wives and mothers must first be educated about and convinced of the benefits of fluoridated salt before it would be widely accepted or used. Women, in turn, would educate family members about specific aspects of their diet and health. These results are similar to but do not echo entirely findings with respect to the acceptability of water fluoridation in this community [13]. If fluoridation of salt were shown to be beneficial for their children, fluoridated salt would be acceptable in this town and most probably in similar communities, too.

### Limitations

This study is limited by its small convenience sample with a truncated income range, and a single rural location. Other limitations are lack of detailed dietary information, especially the daily intake of salt for various age groups in this setting. Caution must be taken when making generalizations especially to other Hispanic or migrant populations, other socioeconomic strata, or different geographic regions. Off-setting some of these limitations, however, is that the study site in California’s Central Valley and the demographic characteristics of participants are reasonably representative of rural US farmworker Latino communities generally [56–57]. Study findings about community beliefs and behaviors, and the implications of these for acceptability and adequate fluoride exposure through salt might therefore be applicable to other vulnerable Latino populations beyond this local context.

As one of few studies that have investigated the acceptability of fluoridated salt for caries prevention in a US community, this study makes a modest but valuable contribution. It is one of very few examinations of how children’s caregivers use domestic salt and understand its properties [78]. Parental perceptions and actions based thereon are highly influential in oral health care seeking for children in minority communities, such as a low-income, rural Hispanic communities, which often have quite distinct oral health beliefs, understandings and responses [26, 53–44,78], as well as a high prevalence of oral disease, especially among young children [12,19–20].

### Fluoridated Salt in the US: Acceptable but not Feasible?

While this study suggests that fluoridated table salt would be acceptable to this and similar small, vulnerable rural Latino communities in the United States, the feasibility of providing fluoridated salt on a mass scale faces many challenges [34]. As noted in the Introduction above, before any future salt fluoridation program could be implemented, considerable nutritional research would first be necessary, regionally if not nationwide. Current fluoride intake levels for people of all ages in large and small, rural and urban, communities would need to be established to ensure that over-exposure to fluoride and its consequences would not occur. Dietary and other surveys would be needed to determine if current nutrient intake, water consumption or oral health and hygiene habits already result in adequate exposure to fluoride. Our study identified individual and community level social and behavioral facilitators and barriers to acceptance of any potential salt fluoridation program in just one type of community.

Barriers to developing a regional or national fluoridated salt program exist at many levels. Among these obstacles are: individual beliefs and values; household purchasing and consumption practices; ability to expand fluoridated salt use beyond the domestic sphere into restaurants, schools, residential institutions (eg., hospitals, prisons, military facilities) and other organizations operating large kitchens (eg., hotels); the availability and nature of supportive community assets including access to and use of fluoridated water supplies; access to and use of other fluoridated products; views and concerns of medical and dental professionals about daily salt (sodium) intake; development and deployment of effective public health education campaigns, and wide scale acceptance of fluoridated salt; monitoring to prevent over-consumption of fluoride; commercial salt and food manufacturing, distribution and marketing processes; and federal regulation and policy adjustments [79].

Despite sustained public health advocacy, there remains considerable suspicion of; resistance to and vocal advocacy against water fluoridation as a caries preventive and the use of fluoride generally [39, 80–81]. Though the scientific evidence for such claims is slim and controversial, many people continue to express a belief that long-term fluoridation leads to deleterious health consequences. Unlike fluoridation of municipal water supplies, which does not easily allow an individual access to a non-fluoridated public source of water, the production of fluoridated salt would not preclude the manufacture and distribution of non-fluoridated salt. Hence, purchase and use of fluoridated salt would be under the control of consumers and could be avoided if so desired. Nevertheless, attempts to introduce fluoridated salt could result in resistance, not just from anti-fluoridation groups but also from segments of the medical and oral health professions. There are legitimate concerns about possible increases in consumption of sodium if fluoridated salt were widely available and marketed as ‘beneficial to health.’ Increased sodium consumption would have undesirable impacts on cardio-vascular and other diseases [75–76]. There is also concern about possible over-exposure to fluoride and the development of dental fluorosis [39, 79, 82] although studies have generally found this to not be a major issue when optimal fluoridation levels are maintained [2, 5, 7, 79]. Some commentators point out that the effectiveness of water fluoridation has waned in recent times compared to its introduction decades ago. Part of the reason for this “halo” effect is the burgeoning of other fluoride-containing products (eg., dentifrices) and foods and drinks in the American diet [39, 51].

Distributors rather than storeowners or managers controlled the timing, quantity and brand of salt delivered to the rural stores in this area of California. Distributors would need to be aware of regional conditions. In a few areas in the United States (eg., specific areas in Colorado), water sources are naturally high in fluoride content, making fluoridation of municipal supplies as a caries (dental decay) preventive unnecessary. Commercial distribution and availability of fluoridated salt in these areas would also need to be restricted, as it is in Mexico where for the same reasons several western states are excluded from participating in that nation’s fluoridated salt program [77]. It can be difficult, however, to confine fluoridated salt to specific locales or patrol its transport across regional boundaries (see p. 54 of the American Dental Association’s publication [5] for an example from Basel, Switzerland).

In Hispanic communities that eschew use of municipal water supplies for drinking or cooking [10–15], over-exposure to fluoride may not be as large a concern as under-exposure in these communities where people are at high-risk for caries. Fluoridated salt could help alleviate under-exposure and boost caries prevention. Commercial salt would need to be monitored regularly to ensure adequacy and appropriateness of fluoride fortification. In Mexico City in 2008, for example, more than 40 brands of fluoridated salt were available, with a fluoride content that ranged widely, from 0 to 485ppm [77; cf. 39]. In other words, fluoride content ranged from complete absence to over-abundance, indicating poor quality control and monitoring. In addition to evaluating fluoride levels in various communal sources, such as water or salt, individual fluoride levels might also need regular monitoring. Those who regularly eat outside their home, drink commercially filtered water, purchase salt at stores outside their region, and use a variety of other fluoride-containing products could also experience variable exposure, making complex the control, monitoring and assessment of fluoride intake and adequacy at the individual let alone the population level [79].

The proportion of processed food in the reported daily diet of this rural Latino population was greater than expected. It has sometimes been suggested that low-income Latino farmworker families generally do not consume commercially processed or packaged foodstuffs, but these results suggest this is not necessarily the case [83]. In this study, commercially prepared items, including dietary staples, were purchased and used on a regular basis. Many of these items—such as bouillon cubes, freeze-dried or ramen noodle soups as well as breads, sausages and hot dogs, pizzas, sports drinks—tend to have a high sodium content [83–86]. In a 2013 report on obesity, The Hispanic Institute argued that the processed food industry should be held accountable for “its complicity in flooding stores with drinks that are too sugary and foods that are too salty and fat” [87].

Salt used in the commercial preparation of canned items is not iodized because chemical reactions that occur during the cooking and preserving of many foods, especially vegetables, lead to undesired color changes rendering the food generally safe to eat but visually unappealing. Use of salt other than sodium chloride can make some pickled or fermented foods unstable or unsafe to consume [35, 88–89]. For similar reasons, it is unlikely that fluoridated salt would be incorporated in the commercial canning and processing of many foodstuffs. This could reduce the extent to which salt consumed on a daily basis in rural Latino households would provide oral health benefits to the population.

At present, commercial manufacturers in the US produce iodized salt on a voluntary rather than a legislated basis [34–35] but there is little incentive for manufacturers to voluntarily add sodium or potassium fluoride to domestic salt. Following very strong public health campaigns in the 1920’s, iodized salt to combat thyroid disease became not only available but normative nationwide in the US [34]. Prior to this, thyroid disease led to extensive disability and death. Without a similar concerted public health campaign about the health impact of caries, which though an extremely common condition is rarely fatal, fluoridation of salt is unlikely to occur. While the amount of sodium or potassium fluoride (NaF or KF) that would need to be added, 200–300mg F per kg of salt, costing about US$0.015 to US$0.030 per capita/year[39], is relatively small and cheap, the technical processes of monitoring and ensuring safety would not necessarily be inexpensive [34]. If monitoring and safety costs were passed along to the consumer, it could result in decreased sales of some brands of domestic table salt because of increased competition from other cheaper (non-fluoridated) brands.

A first hurdle to address, however, could be federal regulation. Existing rules would need to be evaluated and possibly amended to accommodate—or possibly even to mandate—commercial production and distribution of fluoridated salt. The Federal Register [90] describes the current ruling on fluoride and food additives as:

“The Commissioner of Food and Drugs has concluded that it is in the interest of the public health to limit the addition of fluorine compounds to foods (a) to that resulting from the fluoridation of public water supplies, (b) to that resulting from the fluoridation of bottled water within the limitation established in §165.110(d) of this chapter, and (c) to that authorized by regulations (40 CFR part 180) under section 408 of the Act. “[42 FR 14483, Mar. 15, 1977, as amended at 72 FR 10357 Mar. 8, 2007]

Whether importation on a large scale of fluoridated salt from Mexico where it is already commercially produced and distributed, could resolve some feasibility issues is unclear. Globally, a variety of political alliances, trade partnerships and agreements exist regarding the regional manufacture and distribution of various fortified consumable products [34, 91] but none of these agreements was achieved easily or quickly. Existing or revised trade agreements between Mexico and the United States might comprise other barriers or, alternately, might facilitate access to widespread marketing and distribution of this proven caries preventive.

## Conclusion

Fluoridated salt for domestic use would be an acceptable means of delivering a proven preventive for caries under specific circumstances. Low-income rural Latino communities would first have to receive and believe information from trusted, authoritative sources about the safety and benefits of fluoride. Benefits to children would be a powerful motivation for accepting and using this kind of salt product. Equally important from the perspective of the biomedical community, it would have to be demonstrated that fluoridated salt did not lead to an increased consumption of salt, a product with known (deleterious) cardio-vascular effects, especially but not exclusively in adults [78]. Most importantly, fluoridated salt would have to be perceived by the community to taste the same as non-fluoridated salt in order to be accepted for domestic use on a regular basis.

Low-income and rural Latino communities comprise just two types of vulnerable population groups experiencing oral health disparities in the United States. What would benefit these communities would benefit all vulnerable populations with disproportionately high rates of dental decay, especially children five years of age or younger who might not have begun to experience the onset of caries. When water fluoridation is not feasible or present, or not widely used, then salt fluoridation is an effective alternative [34, 41, 51, 91].

Although multifactorial in origin, caries is a largely preventable disease, with fluoride as a major factor in prevention. It is important that fluoride-containing products be developed, be safe, and available on a mass scale, at low cost to consumers, with easy access to purchase and daily use. The feasibility of producing, marketing and distributing fluoridated salt on a commercial scale in the United States, however, is unclear. Many obstacles confront such an enterprise, but the continued exploration of this possibility is warranted by the high prevalence and severity of caries in multiple vulnerable populations, especially children, throughout the nation.
